# Diversity and taxonomic distribution of bacterial biosynthetic gene clusters predicted to produce compounds with therapeutically relevant bioactivities

**DOI:** 10.1093/jimb/kuad024

**Published:** 2023-08-31

**Authors:** Max L Beck, Siyeon Song, Isra E Shuster, Aarzu Miharia, Allison S Walker

**Affiliations:** Department of Chemistry, Vanderbilt University. 1234 Stevenson Center Lane, Nashville, TN 37240, Untited States; Department of Chemistry, Vanderbilt University. 1234 Stevenson Center Lane, Nashville, TN 37240, Untited States; Department of Chemistry, Vanderbilt University. 1234 Stevenson Center Lane, Nashville, TN 37240, Untited States; Department of Chemistry, Vanderbilt University. 1234 Stevenson Center Lane, Nashville, TN 37240, Untited States; Department of Chemistry, Vanderbilt University. 1234 Stevenson Center Lane, Nashville, TN 37240, Untited States; Department of Biological Sciences, Vanderbilt University. VU Station B, Box 35-1634, Nashville, TN 37235, Untited States

**Keywords:** Natural products, Genome mining, Machine learning

## Abstract

Bacteria have long been a source of natural products with diverse bioactivities that have been developed into therapeutics to treat human disease. Historically, researchers have focused on a few taxa of bacteria, mainly *Streptomyces* and other actinomycetes. This strategy was initially highly successful and resulted in the golden era of antibiotic discovery. The golden era ended when the most common antibiotics from *Streptomyces* had been discovered. Rediscovery of known compounds has plagued natural product discovery ever since. Recently, there has been increasing interest in identifying other taxa that produce bioactive natural products. Several bioinformatics studies have identified promising taxa with high biosynthetic capacity. However, these studies do not address the question of whether any of the products produced by these taxa are likely to have activities that will make them useful as human therapeutics. We address this gap by applying a recently developed machine learning tool that predicts natural product activity from biosynthetic gene cluster (BGC) sequences to determine which taxa are likely to produce compounds that are not only novel but also bioactive. This machine learning tool is trained on a dataset of BGC-natural product activity pairs and relies on counts of different protein domains and resistance genes in the BGC to make its predictions. We find that rare and understudied actinomycetes are the most promising sources for novel active compounds. There are also several taxa outside of actinomycetes that are likely to produce novel active compounds. We also find that most strains of *Streptomyces* likely produce both characterized and uncharacterized bioactive natural products. The results of this study provide guidelines to increase the efficiency of future bioprospecting efforts.

**One-Sentence Summary:**

This paper combines several bioinformatics workflows to identify which genera of bacteria are most likely to produce novel natural products with useful bioactivities such as antibacterial, antitumor, or antifungal activity.

## Graphical Abstract

**Graphical Abstract:**
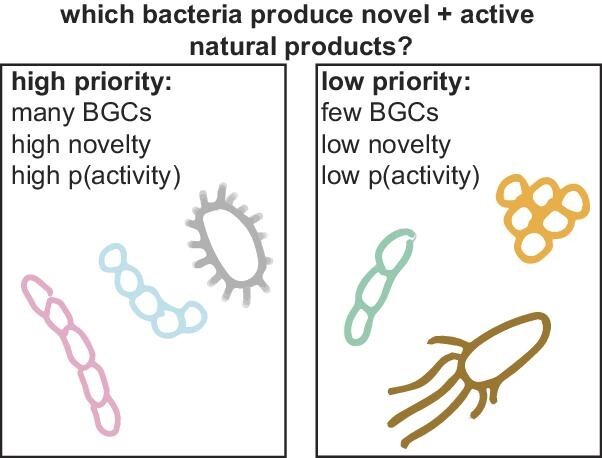
Multiple bioinformatics methods are combined to identify the most promising bacterial taxa for genome mining—those that contain many BGCs that are likely to be novel and produce active natural products.

## Introduction

Natural products are an especially rich source of molecules with therapeutic potential. Many therapeutics used to treat human disease originate from natural products—23.5% of FDA-approved drugs are either natural products or chemically modified natural product derivatives (Newman and Cragg, 2020). One of the major challenges in natural product discovery is rediscovery of known compounds, especially when isolating compounds from historically popular sources of natural products, such as *Streptomyces* (Kong et al., 2011). Previous studies have surveyed biosynthetic diversity across the bacterial taxonomy and found that there are many families of biosynthetic gene clusters (BGCs) with no known product, even in well-mined genera such as *Streptomyces* and other actinomycetes (Jensen et al., 2014; Schorn et al., 2016; Chevrette and Handelsman, 2021; Gavriilidou et al., 2022; Gonzalez-Salazar et al., 2023). A study of the chemical diversity throughout the history of natural product discovery showed similar results—while the average novelty of structures discovered has decreased over time there remains a slow and steady discovery of novel scaffolds (Pye et al., 2017). One bioinformatics study used various BGC clustering methods and rarefaction analysis to estimate that there are over 90 000 gene cluster families (GCFs) present in bacterial genomes, most of which have yet to be sequenced (Gavriilidou et al., 2022). However, we still do not know how many of these uncharacterized BGCs will produce a compound with a therapeutically relevant bioactivity. It is possible that many of these uncharacterized clusters produce compounds with other activities, such as siderophores, ionophores, or signaling molecules, all of which are common natural product functions (Lyon and Muir, 2003; Swayambhu et al., 2021). It has been suggested that all broad-spectrum antibiotics produced by *Streptomyces* have already been discovered (Lewis, 2020). An alternative explanation is that undiscovered antibiotics are rarely or never expressed under lab growth conditions. To determine the reason for the lack of discovery of novel antibiotics from *Streptomyces* it is essential to be able to identify how likely uncharacterized BGCs are to produce compounds with various bioactivities.

It will likely be easier to discover novel bioactive compounds from bacterial taxa that have not been the focus of previous natural product discovery campaigns. There have been several reported discoveries from underexplored taxa such as *Salinispora* and other marine actinomycetes (Jensen et al., 2014; Kim et al., 2021) and *Couchioplanes* (McClung et al., 2022). However, it is unclear what the optimal criteria are for selecting taxa for natural product discovery campaigns. This problem is not trivial because there are 19 153 genera of bacteria in the Genome Taxonomy Data Base (GTDB) (Parks et al., 2022) and likely many additional genera that have yet to be cultured (Hug, 2018). It is important to prioritize those that are likely to produce novel natural products with promising bioactivities to make genome mining more efficient. Modern genome mining techniques can help determine which taxa are the optimal targets for genome mining.

**Fig. 1.**
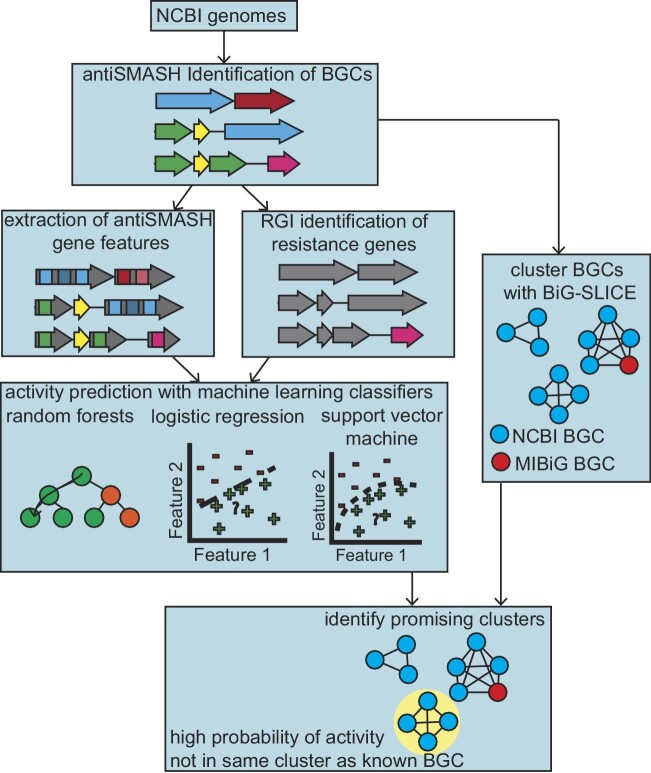
Diagram illustrating our workflow for this work. BGCs are first identified by antiSMASH. antiSMASH and RGI annotations are used as inputs to machine learning models that predict the activity of the BGC's product. We then combine these results with BiG-SLiCE analysis to identify GCFs that are likely to produce novel and active compounds.

There are several existing methods for predicting the activity of natural products from the sequence of the BGCs that produce them (Hannigan et al., 2019; Skinnider et al., 2020; Walker and Clardy, 2021). These methods can be used to prioritize BGCs that are likely to produce natural products with therapeutically relevant bioactivities. Here, we apply one of those methods, which we previously developed (Walker and Clardy, 2021) to 583 922 BGCs from 110 246 genomes to identify genera that are likely to produce multiple compounds with antibacterial, antitumor, or antifungal activities. Our method predicts natural product bioactivities using three machine learning classifiers: random forests, logistic regression, and support vector machines that have been trained on BGC-natural product activity pairs. The features of the BGC used for training are the protein domain annotations as determined by antiSMASH (Blin et al., 2019) and resistance gene annotations as determined by resistance gene identifier (RGI) (Alcock et al., 2020). Therefore, the model relies on a variety of aspects of the BGC to make its predictions, such as biosynthetic capacity and presence of transporters and other resistance genes. Separate classifiers are trained for different activities because natural products may have multiple activities. These classifiers can predict natural product activity with accuracies as high as 80% (Walker and Clardy, 2021). We combined our predictions with clustering analysis performed using BiG-SLiCE (Kautsar et al., 2021). Characterized BGCs from the MIBiG (Terlouw et al., 2023) database were included in the BiG-SLiCE analysis to enable us to assess the novelty of different BGCs—BGCs that do not cluster with characterized BGCs are more likely to produce novel compounds (Fig. 1). We find that our method predicts that there are likely still many undiscovered antibacterial and other bioactive compounds produced by well-mined actinomycetes, such as *Streptomyces*, although they may have a narrow spectrum of activity. In addition, we identify several rare actinomycetes such as *Polymorphospora, Desertactinospora, Herbidospora* and bacteria outside of actinomycetes such as *Pseudomonas E, Burkholderia, Bradyrhizobium*, and *Bacillus A* that are promising sources of bioactive natural products with a lower risk of compound rediscovery. Based on these results, we recommend the careful dereplication of known compounds for any genera that we identified as likely to produce known compounds and suggest that the community should focus primarily on taxa that may produce exclusively novel compounds.

## Materials and Methods

### Determining Taxonomy

Taxonomy was determined using GTDB (Parks et al., 2022), for genomes not present in GTDB, we used the GTDB-Tk version 2 to assign taxonomy (Chaumeil et al., 2022).

### BGC Identification and Activity Predictions

Genomes in genbank format were obtained from the National Center for Biotechnology Information (NCBI) RefSeq database (O'Leary et al., 2016), a full list of genome accessions is available in Table S1. BGCs were identified using antiSMASH version 5 (Blin et al., 2019). antiSMASH was run with the fullhmmer option to add PFAM annotations, which are needed for activity prediction and the genefinding-tool prodigal option to annotate genes for genomes that were not already annotated. antiSMASH cluster genbank files were converted to fasta format using a custom script, convert_antiSMASH_to_fasta.py, available at https://github.com/aswalker-lab/BioactiveNP_Distbution. RGI version 5 (Alcock et al., 2020) was run on the BGC fasta files used to identify resistance genes. The antiSMASH and RGI results were then used to predict the bioactivities of the products of the BGCs using an activity prediction method we previously developed (Walker and Clardy, 2021). As described in our previous publication, counts of protein family (PFAM) domains, CDS motifs, smCOG annotations, polyketide or nonribosomal peptide monomer predictions, and resistance gene annotations are represented as vector which is used to train the random forest, logistic regression, and support vector machine classifiers. We used the hyperparameters and training data described in (Walker and Clardy, 2021) to build and train our models. The predictions were run with the no_SSN option and with a random seed of 0.

### Clustering BGCs into GCFs

BGCs were downloaded from the MIBiG database version 3.1 (Terlouw et al., 2023) and converted for input to BiG-SLiCE using the generate_antismash_gbk.py script from the BiG-SLiCE (Kautsar et al., 2021) github (https://github.com/medema-group/bigslice/blob/master/misc/generate_antismash_gbk/generate_antismash_gbk.py). We followed a procedure described in Gavriilidou et al. (2022) to cluster the BGCs identified by antiSMASH and BGCs from the MIBiG database. BiG-SLiCE output was reclustered into GCFs using an l2-norm distance threshold of 0.4 as suggested in Gavriilidou et al. (2022) with slight modifications made to the l2-norm clustering script to output additional information used in our downstream analysis. We obtained the original version of the l2-norm script from https://github.com/medema-group/bigslice/blob/master/misc/useful_scripts/perform_l2norm_clustering.py and our modified version is available: https://github.com/aswalker-lab/BioactiveNP_Distbution.

### Downstream Analysis of GCFs and BGCs

Each GCF was classified as containing at least one MIBiG BGC or not. Our machine learning method predicts the following activities: antibacterial, anti-gram-positive bacteria, anti-gram-negative bacteria, antitumor or antifungal (referred to as antieukaryotic activity in this work), antifungal, and antitumor. Probabilities for each class of prediction were averaged across the three different classifiers (random forest, logistic regression, and support vector machine). BGCs were predicted as likely to produce an active compound if this averaged probability was greater than 0.5. We then counted the number of predicted active BGCs in each genome for each activity prediction and characterized them as being the same GCF as an MIBiG BGC or not. We also classified each GCF as likely to produce active compounds by averaging the activity probability across all BGCs in the GCF, again using 0.5 as a cutoff.

## Results and Discussion

### Identifying Taxa That Harbor Many Novel and Bioactive GCFs

We first set out to determine the total number of GCFs that are likely to produce novel active compounds from each genus across the genomes in our data set. We first downloaded a selection of 110 246 genomes from the NCBI RefSeq database (O'Leary et al., 2016). We then used antiSMASH (Blin et al., 2019) to identify biosynthetic gene clusters in these genomes. antiSMASH is also used to identify various BGC annotations that our machine learning model uses as features for its predictions—these include protein family (PFAM) domains, CDS motifs, smCOGs, and polyketide and nonribosomal peptide monomer predictions. Each BGC was analyzed with the RGI (Alcock et al., 2020) to annotate antibiotic resistance genes present in the BGC. The output of antiSMASH and RGI were used as input to our machine learning models to predict the probability that the BGC produces a compound with antibacterial, anti-gram-positive, anti-gram-negative, antitumor or antifungal (subsequently referred to as antieukaryotic), antitumor, and antifungal activity with three types of machine learning classifiers as previously described (Walker and Clardy, 2021) (Fig. 1). The three classifiers are random forest, logistic regression, and support vector machines, which are commonly used classifiers for binary classification problems. These classifiers are built using the Python module scikit-learn (Pedregosa et al., 2011) and we use our previously reported optimized hyperparameters for each prediction task (Walker and Clardy, 2021). Each classifier is independently trained for each activity prediction task on a dataset of BGCs that have been annotated as producing product with or without the activity of interest based on literature reports. These classifiers can then be used to predict if a novel BGC will produce a compound with that activity. In total, we predicted activity for 583 922 BGCs.

**Fig. 2.**
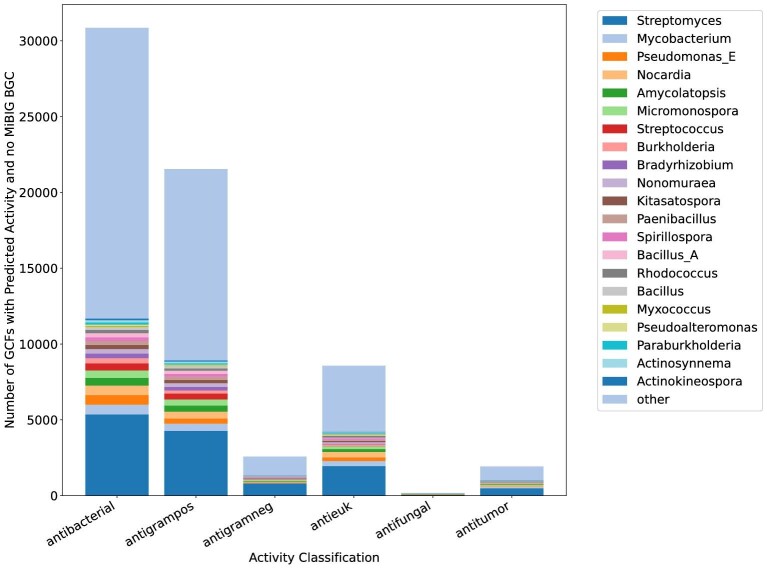
Predicted number of GCFs that produce active compounds per genera. Only genera that were in the top 10 of one of the activity predictions are shown, all other genera are contained in the other category. Note that the top 10 genera are different for different activities, so there are more than 10 genera shown.

We then used BiG-SLiCE (Kautsar et al., 2021) to cluster these BGCs into gene cluster families (GCFs) and reclustered them based on their l2-norm with a distance threshold of 0.4 using a procedure described in (Gavriilidou et al., 2022). Clusters from MIBiG version 3 (Terlouw et al., 2023) were also included in the BiG-SLiCE analysis. To determine if a BGC is likely to produce a known compound, we checked if it was in the same GCF as one or more of the MIBiG BGCs. It is important to note that MIBiG does not contain BGCs for all known natural products, therefore some of the GCFs that do not contain MIBiG BGCs probably also produce known natural products. We then classified both BGCs and GCFs based on if they were likely to produce an active compound using our machine learning classifications for each activity. For each genus, we counted the total number of GCFs likely to produce compounds with each activity, splitting the GCFs into two groups—those that did not contain an MIBiG BGC and those that did. GCFs that are likely to produce an active compound and that do not contain an MIBiG BGC will be the most promising targets for genome mining efforts because they are more likely to produce novel active compounds. For each activity, we ranked all genera by the number of GCFs contained in at least one genome for that genera that did not contain an MIBiG BGC and were predicted to produce active compounds. We identified the top 10 genera for each activity. The number of predicted GCFs for each of these genera, as well as genera outside of the top 10 is shown in Fig. 2. We also determined the top 25 genera ranked by number of GCFs without an MIBiG BGC predicted to produce antibiotic or antieukaryotic compounds. These data are shown in Figs. S1 and S2, respectively, and a full list of all genera for all activities are available in Tables S2–7.

Genera containing many of these GCFs are likely to produce uncharacterized active natural products. We found that *Streptomyces* have by far the largest number of predicted uncharacterized GCFs that produce antibiotics, with 5 358 GCFs for antibacterial activity (Figs. 2 and S1) and 1 954 GCFs for antieukaryotic activity (Figs. 2 and S2). This is consistent with other reports of *Streptomyces* containing an extreme diversity of BGCs (Gavriilidou et al., 2022). The genus with the second largest number of uncharacterized GCFs predicted to produce an antibiotic is *Mycobacterium* with 641 GCFs. *Nocardia* was predicted to contain the second highest number of GCFs that are likely to produce a novel compound with antitumor or antifungal activity with 356 GCFs. The remaining genera that have a large number GCFs that likely produce novel active compounds include other actinomycetes that have been extensively sequenced such as *Amycolatopsis* and *Micromonospora* as well as other well-sequenced gram-positive bacteria such as various bacilli (e.g., *Bacillus A* and *Paenibacillus*) and gram-negative bacteria such as *Pseudomonas E, Burkholderia*, and rhizobia bacteria (e.g., *Bradyrhizobium* and *Rhizobium*). The biosynthetic potential of many of these taxa has been noted previously and some secondary metabolites from these taxa have been characterized (Gross and Loper, 2009; Adamek et al., 2018; Braesel et al., 2018; Kunakom and Eustaquio, 2019; Hifnawy et al., 2020; Steinke et al., 2021; Bach et al., 2022). Still, the number of GCFs containing MIBiG BCGs for these taxa are significantly lower than for *Streptomyces* (Figs. S1 and S2) suggesting that while these taxa may not contain the total biosynthetic diversity of *Streptomyces*, fewer of their natural products have been characterized. While some genera, such as *Streptomyces*, are predicted to produce a similar percentage of the total compounds with different activities, others are more variable. For example, *Streptococcus* and *Bradyrhizobium* are both predicted to harbor a larger percentage of the predicted antibacterial-producing GCFs (1.5 and 1.0%, respectively) than GCFs predicted to produce compounds with antieukaryotic activity (0.14 and 0.61%). These variations could be due to different selective pressures on these bacteria due to differences in environment or lifestyle.

One limitation of this analysis is that there is an extreme bias in how many sequenced genomes there are per genera in the NCBI database. The NCBI database is biased toward abundant organisms that are easy to isolate such as *Streptomyces* (1186 genomes in our dataset) and *Bacillus A* (1208) as well as genera that include important human pathogens such as *Mycobacterium* (2890), *Staphylococcus* (7228), and *Pseudomonas E* (2410). The large number of sequenced genomes for these genera likely partially accounts for their high ranking in this analysis.

### Determining How Many BGCs Per Genome are Likely to Produce Novel and Bioactive Compounds

**Fig. 3.**
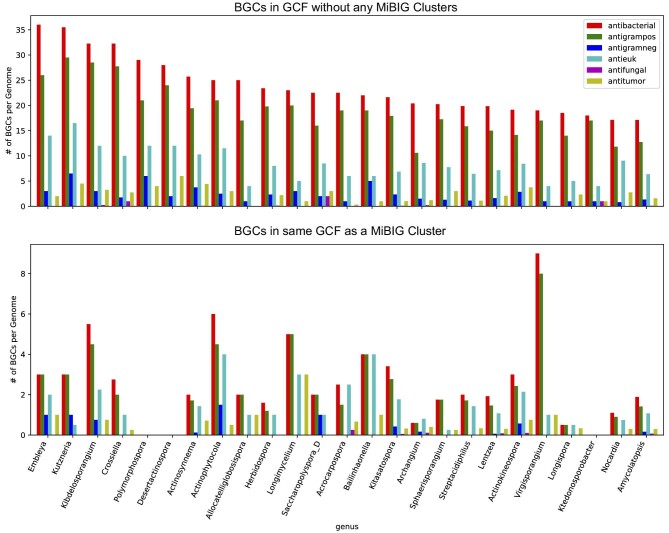
Average predicted number of BGCs that produce active compounds per genome, ranked by the number of BGCs predicted to produce a compound with antibacterial activity that are not in the same GCF as an MIBiG cluster. The top graph shows the number of BGCs that are not in the same GCF as an MIBiG cluster, and are therefore likely to produce a novel compound. Colors indicate the predicted activities: antibacterial (red), anti-gram positive (green), anti-gram negative (blue), antieukaryotic (antitumor or antifungal, cyan), antifungal (purple), and antitumor (yellow).

**Fig. 4.**
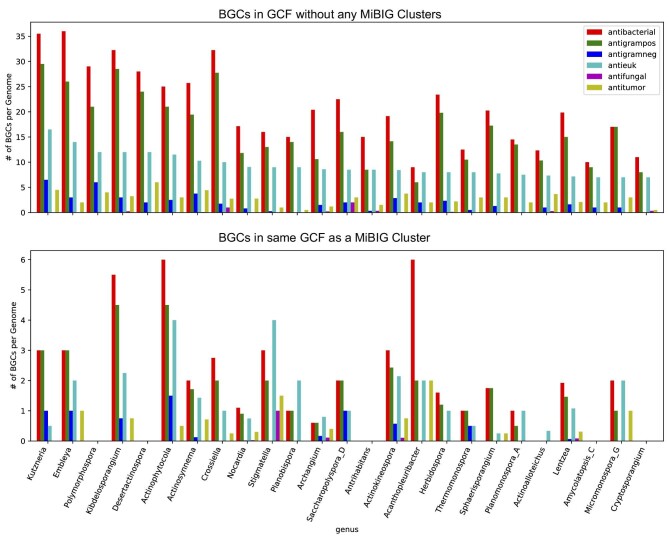
Average predicted number of BGCs that produce active compounds per genome, ranked by the number of BGCs predicted to produce a compound with antitumor or antifungal activity that are not in the same GCF as an MIBiG cluster. The top graph shows the number of BGCs that are not in the same GCF as an MIBiG cluster, and are therefore likely to produce a novel compound. Colors indicate the predicted activities: antibacterial (red), anti-gram positive (green), anti-gram negative (blue), antieukaryotic (antitumor or antifungal, cyan), antifungal (purple), and antitumor (yellow).

To account for the bias in the NCBI database discussed above we also analyzed how many BGCs predicted to produce active compounds are present per genome. This analysis will also allow us to determine how the number of BGCs that likely produce a novel compound compare to the number of BGCs that are likely to produce known compounds. Species that produce only novel compounds will not require extensive dereplication efforts. The top 25 genera ranked by expected number BGCs per genome that produce novel compounds with antibacterial or antieukaryotic activity are shown in Figs. 3 and 4, respectively, and the full ranking by each activity is available in Table S8–13. In striking contrast to the analysis of the total number of novel GCFs per genus, in this analysis *Streptomyces* are not particularly distinct, ranking 36th and 46th in number of BGCs not in the same GCF as an MIBiG BGC expected to produce antibacterial or antieukaryotic compounds, respectively. Rare actinomycetes such as *Polymorphospora, Desertactinospora*, and *Herbidospora* are predicted to have slightly more BGCs per strain that produce novel antibiotic or antieukaryotic compared to *Streptomyces*. This is consistent with previous observations of the large number of BGCs present in rare actinomycetes (Jensen et al., 2014; Schorn et al., 2016; Gonzalez-Salazar et al., 2023). This result suggests that *Streptomyces* are not unique as prodigious producers of bioactive natural products.

While our analysis suggests that there are still many bioactive compounds produced by *Streptomyces* that have yet to be discovered, we also observe that, on average, each *Streptomyces* to is expected to produce three known antibiotics and two known antieukaryotic compounds. This is also true of several other actinomycetes genera such as *Embelya, Kurtzneria*, and *Kibdelosporangium*, which are all expected to produce multiple characterized and novel compounds (Figs. 3 and 4). Without rigorous dereplication and annotation of all unknown metabolites it is likely that novel active compounds produced by these genera could be missed if there are known active compounds produced in larger quantities. Meanwhile, strains of the rare actinomycetes discussed above are expected to predict few or no known products. The non-actinomycetes discussed above contain fewer BGCs per genome than the rare actinomycetes but are still likely to produce novel active compounds. For example, *Pseudomonas E* is predicted to have four novel antibiotic-producing BGCs per genome and *Bradyrhizobium* is predicted to have five novel antibiotic-producing BGCs per genome (Table S8). Based on this result, we suggest that the natural products community should put effort into identifying environments where these rare actinomycetes are present and into developing isolation techniques that preferentially isolate these actinomycetes over *Streptomyces*. We also recommend the use of modern dereplication methods when dealing with *Streptomyces* or other strains likely to produce known metabolites (Bradshaw et al., 2001; Dieckmann et al., 2005; Mohimani et al., 2018).

### Limitations of Analysis

There are several limitations of the analysis described above. First, our machine learning method is not perfectly accurate at predicting bioactivites. Antibacterial activity classification is the most accurate, reaching up to 80% accuracy but prediction of antifungal activity is lower at 57%. In addition, accuracy decreases for BGCs with a higher degree of novelty (Walker and Clardy, 2021). Another limitation is that many of the genomes included in our analysis are not complete and may be composed of many short contigs. As a result, a single BGC may be split across multiple contigs and either not identified or identified as multiple BGCs. This will affect the accuracy of activity classification, clustering, and number of BGCs counted per genome. For some of the genera studied, there were only one or two genomes present in the data set and therefore the number of BGCs may not be representative of the whole genus. Therefore, the numbers presented in this study should be considered as an estimate of the capacity of organisms to produce bioactive natural products and not an exact calculation.

## Conclusions

In this work we analyzed how many novel and active natural products different genera of bacteria are likely to produce using BGC clustering and machine learning activity prediction. We find that there are likely many undiscovered bioactive natural products, which is consistent with previous reports that identified significant untapped biosynthetic diversity. We find that even well mined taxa such as *Streptomyces* are likely to produce undiscovered bioactive natural products. However, genome mining in *Streptomyces* may be more challenging because each strain is expected to also produce known active compounds which will have to be dereplicated. Therefore, we recommend that bioprospecting efforts focus on rare actinomycetes and non-actinomycetes that have many BGCs likely to produce novel active compounds. Fewer of these organisms have been isolated and sequenced compared to *Streptomyces* and so we also recommend that the scientific community focus on developing isolation protocols that are more selective for these organisms. Our results suggest that such efforts will result in the discovery of a significant number of novel bioactive compounds.

## Supplementary Material

kuad024_Supplemental_Files
