# Supplementary material for: Diversity and taxonomic distribution of bacterial biosynthetic gene clusters predicted to produce compounds with therapeutically relevant bioactivities
Source: J Ind Microbiol Biotechnol. 2023 Aug 31;50(1):kuad024. doi: 10.1093/jimb/kuad024 (PMC10548851; doi:10.1093/jimb/kuad024)
Supplement: kuad024_Supplemental_Files [file kuad024_supplemental_files.zip › SI Figures.docx]

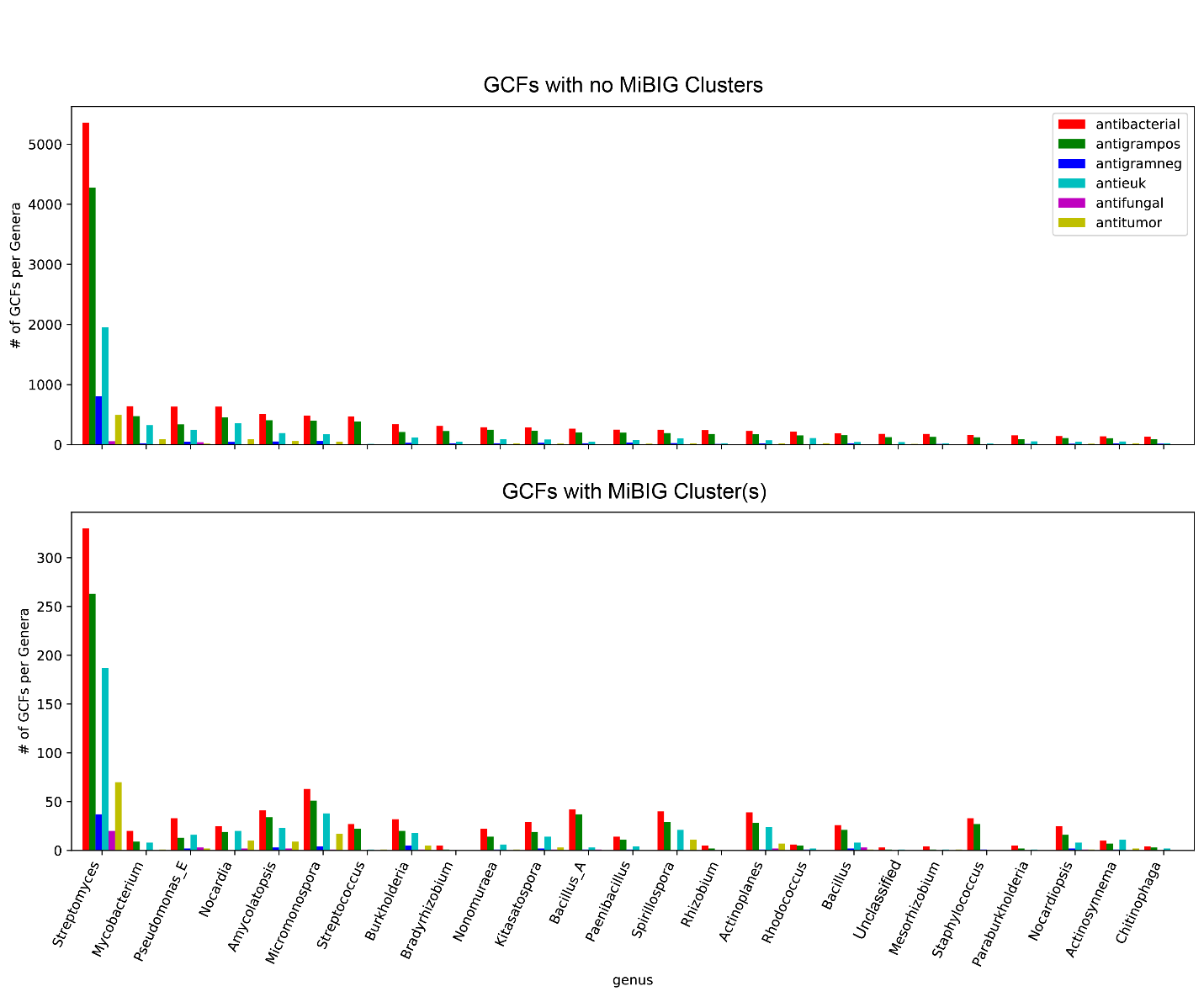


**Figure S1.** Predicted number of GCFs that produce active compunds per genera, ranked by the number of GCFs predicted to produce an antibiotic compound that do not contain an MiBIG BGC. The top graph shows the number GCFs that do not contain an MiBIG BGC and the bottom graph shows the number of GCFs that do contain an MiBIG BGC. Colors indicate the predicted activities: antibacterial (red), anti-gram positive (green), anti-gram negative (blue), antieukaryotic (antitumor or antifungal, cyan), antifungal (purple), antitumor (yellow).


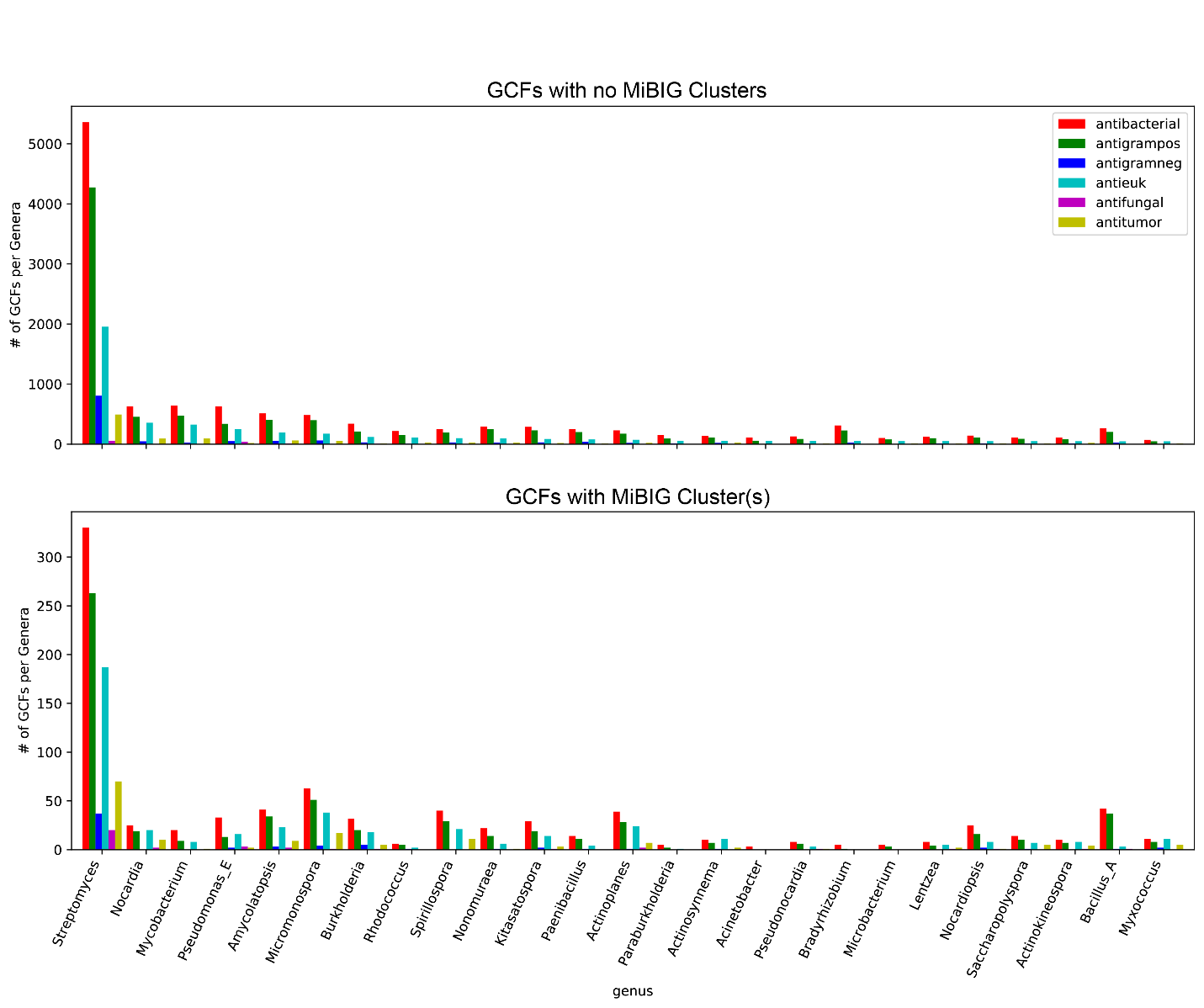


**Figure S2.** Predicted number of GCFs that produce active compunds per genera, ranked by the number of GCFs predicted to produce a compound with antitumor or antifungal activity that do not contain an MiBIG BGC. The top graph shows the number GCFs that do not contain an MiBIG BGC and the bottom graph shows the number of GCFs that do contain an MiBIG BGC. Colors indicate the predicted activities: antibacterial (red), anti-gram positive (green), anti-gram negative (blue), antieukaryotic (antitumor or antifungal, cyan), antifungal (purple), antitumor (yellow).
